# QuorUM: An Error Corrector for Illumina Reads

**DOI:** 10.1371/journal.pone.0130821

**Published:** 2015-06-17

**Authors:** Guillaume Marçais, James A. Yorke, Aleksey Zimin

**Affiliations:** IPST, University of Maryland, College Park, MD, USA; University of North Carolina at Charlotte, UNITED STATES

## Abstract

**Motivation:**

Illumina Sequencing data can provide high coverage of a genome by relatively short (most often 100 bp to 150 bp) reads at a low cost. Even with low (advertised 1%) error rate, 100 × coverage Illumina data on average has an error in some read at every base in the genome. These errors make handling the data more complicated because they result in a large number of low-count erroneous *k*-mers in the reads. However, there is enough information in the reads to correct most of the sequencing errors, thus making subsequent use of the data (e.g. for mapping or assembly) easier. Here we use the term “error correction” to denote the reduction in errors due to both changes in individual bases and trimming of unusable sequence. We developed an error correction software called QuorUM. QuorUM is mainly aimed at error correcting Illumina reads for subsequent assembly. It is designed around the novel idea of minimizing the number of distinct erroneous *k*-mers in the output reads and preserving the most true *k*-mers, and we introduce a composite statistic π that measures how successful we are at achieving this dual goal. We evaluate the performance of QuorUM by correcting actual Illumina reads from genomes for which a reference assembly is available.

**Results:**

We produce trimmed and error-corrected reads that result in assemblies with longer contigs and fewer errors. We compared QuorUM against several published error correctors and found that it is the best performer in most metrics we use. QuorUM is efficiently implemented making use of current multi-core computing architectures and it is suitable for large data sets (1 billion bases checked and corrected per day per core). We also demonstrate that a third-party assembler (SOAPdenovo) benefits significantly from using QuorUM error-corrected reads. QuorUM error corrected reads result in a factor of 1.1 to 4 improvement in N50 contig size compared to using the original reads with SOAPdenovo for the data sets investigated.

**Availability:**

QuorUM is distributed as an independent software package and as a module of the MaSuRCA assembly software. Both are available under the GPL open source license at http://www.genome.umd.edu.

**Contact:**

gmarcais@umd.edu.

## Introduction

While second generation sequencing technologies have progressed tremendously and offer ever longer reads with low overall sequencing error rate, correcting errors in reads remains a desired pre-processing step in *de novo* genome assembly. Most current assembly software use the de Bruijn graph representation as major part of the assembly process [1–4]. The de Bruijn graph made from raw reads (i.e. not error corrected) is likely larger and more complicated, making the assembly process more difficult and error-prone. In general, error correcting the reads leads to assemblies with longer contiguous sequences and fewer misassemblies [5, 6].

In this paper we present a new error correction procedure and software package, named QuorUM (Quality Optimized Reads from the University of Maryland), that reduces errors by both trimming and changing individual bases in Illumina reads. It is targeted at improving genome assembly. QuorUM works as a stand-alone program and is also a component of the assembler MaSuRCA [7]. It is designed to efficiently work the large data sets produced by today’s high throughput sequencing machines. On our system, it checks approximately 1 billion bases per hour per core (2GHz Intel or AMD) and it scales nearly linearly with multiple cores. We evaluate the error correction skill of QuorUM and compare it to other error correction programs on three genomes that have Illumina reads and have high quality reference assemblies. The other programs used for comparison are: HiTec [8], Echo [9], Coral [10], SGA [11], Racer [12], Musket [13] and Quake [14].

### Trimming

In our view, trimming is an integral part of error correction. The distribution of sequencing errors in the reads is complex, and for some percentage of the reads, the sequence beyond a certain point contains too many errors to be corrected or, even worse, does not correspond to any sequence in the original genome. It is important to trim those reads to avoid misassemblies [5]. Indeed, in the programs that trim, such as Quake [14] there is generally no option to turn this feature off.

Trimming can be viewed as an error reduction step. Since for Illumina sequencing, the base quality degrades toward the 3’ ends of the reads one can reduce the overall error rate in the data by trimming the reads by a fixed amount. One can also trim based on the quality values reported by the sequencing machine. Although these simple trimming schemes will reduce the number of erroneous bases, they still leave many errors in the reads and needlessly discard a lot of valid sequence. Trimming too aggressively will result in fragmented assemblies.

QuorUM does not trim a fixed amount nor trim below a certain quality score. Rather, it detects when a region is “questionable” in the sense of requiring too many base changes. It trims off such questionable regions.

### Not eliminating rare *k*-mers

Random sequencing errors result in spurious *k*-mers, which, with high probability, occur only rarely in the reads. A common approach to error reduction in graph-based assembly techniques is to eliminate rare *k*-mers from the *k*-mer database [3, 15–17]. In that approach, one determines a threshold and eliminates from the reads each *k*-mer whose multiplicity in the reads is below that threshold.

Setting such a threshold works well when there is uniform coverage or, at least, when there is high coverage throughout the entire genome. In practice, with second generation sequencing, parts of the genome will have low or zero coverage. The method with a threshold will accurately correct the regions with high coverage and will convert low coverage regions to zero coverage regions thus creating gaps in the assembly.

A central theme of QuorUM is to preserve low coverage regions when possible by not correcting any bases in low coverage regions unless there is evidence that the base is definitely erroneous and we know what the replacement base should be.

### Other approaches

Some other approaches are not based on the multiplicities of *k*-mers in the reads. For example, Coral and Echo use multiple alignment of the reads and statistical models of sequencing to correct misaligned bases. HiTec uses a suffix array to find and correct potentially erroneous bases. Coral, Echo, HiTec, SGA and Racer only attempt to make base substitutions, while Quake, Musket and QuorUM will also trim reads. See [18] for a brief survey of methods used to correct reads.

Finally we note that QuorUM only corrects substitution errors, not insertions and deletions (except via trimming). It is well suited for correcting reads sequenced using Illumina technology [19], where the substitutions errors are the most common.

## Methods

QuorUM is based on heuristic approach designed by examining the properties of Illumina sequencing data. One difference between QuorUM and most other published error correctors is that QuorUM does not assume that low count *k*-mers are likely to be erroneous. One cannot make such an assumption if the error-corrected reads are to be used for assembly because of high local variability of the coverage of a genome by Illumina reads due to sequencing and library construction biases. Instead QuorUM assesses the validity of a *k*-mer in a read using a variety of statistics, including the number of occurrences in the reads, the quality value of the bases, and the continuity of the coverage. QuorUM trims off any end of a read that otherwise seems to require numerous corrections.

We based our approach on the following properties of the data:

1. the base quality is generally high at 5’ end and it deteriorates toward 3’ end for most reads
2. it is quite common to have the same substitution error in multiple reads or an error in high quality (*q* > 30) base; thus neither the number of occurrences of a *k*-mer in the reads nor the quality score alone can be used as a reliable way to identify correct or erroneous *k*-mers

We designed the QuorUM error reduction algorithm to be conservative: unless we have evidence that a base is erroneous and we know with strong likelihood what the unique substitution is, we do not change the base.

### Two sets of *k*-mers in reads

Using the *k*-mer counter Jellyfish [20], we create a list or hash of all the *k*-mers that occur in the reads and record each *k*-mer’s ***count***, that is, the number of times that the *k*-mer occurs in the set of reads. The *k*-mer and its reversed complement are identified, meaning that the count of a *k*-mer *m* is the number of occurrences of *m* plus the number of occurrences of the reverse complement of *m*. We mark the *k*-mers for which there is at least one occurrence in the reads in which all bases in the *k*-mer have at least quality *q* and we call these reliable. We call *q* the quality threshold and is set to 5 by default. We found that the algorithm is not sensitive to this choice in a significant way: the results were similar for thresholds between 4 and 8. Let *R* (Reliable) denote the set of reliable *k*-mers and *A* (All) is the set of all *k*-mers.

### Start procedure

We begin correcting a read by creating a string *S*, initially consisting of the string of bases in the read. We will possibly modify the string *S* to obtain the corrected read. We choose as a starting point for error detection the first *k*-mer in the read with a count of at least 3. We call it the ***anchor*** *k*-mer. (If none exists, the read is discarded.) By definition, we assume that the anchor *k*-mer is valid, i.e. all bases in it are correct. We proceed forward and backward from this *k*-mer. We only describe the forward procedure below, that is, correcting while moving toward the 3’ end. The backward procedure is essentially identical.

### Evaluating a base

At each iteration, after accepting a *k*-mer *m* as valid, we shift one base in the forward direction, and evaluate the correctness of that base as follows. Let *b* denote the next base in *S* beyond m. Let *m*′ denote the (*k*−1)-suffix of *m* and *m*′*x* denote the *k*-mer consisting of *m*′ with some base *x* appended. For example *m*′*b* will denote the *k*-mer in *S*. (Note that it may differ from the corresponding *k*-mer in the read if a recent substitution has been made). In the following, *c* corresponds to the count of the *k*-mer *m*, the *k*-mer prior to the one under consideration. Also, we always consider only the non-zero counts of the *m*′*x* (with *x* in {*A*, *C*, *G*, *T*}) in the reliable database *R*, if there are any. Only if all the counts are 0 in the *R* database do we consider the *A* database. In other words, reliable *k*-mers always trump others.

### Case 1: Unique continuation

If there is a unique choice of the base *x* for which *m*′*x* has non-zero count, we accept *m*′*x* and *x* becomes a base in *S*. Of course that might mean there is no change since *x* might equal *b*. If there is no unique continuation, proceed to Case 2.

### Case 2: Count threshold

If *m*′*b* has a count greater than some integer threshold *T*, we accept *m*′*b* and no change is made to *S*. Otherwise, proceed to Case 3. The program chooses *T* based on the local estimate of coverage. When the count of *m*′*b* is greater or equal to *T*, then the probability of *b* being the wrong base is less than 1 in a million. Notice that we have threshold above which *k*-mers are considered valid, below which *k*-mers might be valid.

### Case 3: Continuity of coverage

We choose the base *x* such that the count of *k*-mer *m*′*x* is closest to *c* (the count of the previous *k*-mer), provided that only one such base exists. Otherwise, if there is not a unique choice, we proceed to case 4. As in case 1, *x* and *b* might be equal and no change is made.

### Case 4: No change

If none of the above apply, make no change if the count of *m*′*b* is non-zero. If it is zero, trim the read at the current base.

We repeat this process until we come to the end of the read or until the read is trimmed.

In making base changes, we do not allow more than *M* substitutions in a window of *W* bases. In that case, we trim the read just prior to the first change in the window *W*. The default values are *M* = 3 and *W* = 10.

## Results

We only use real Illumina data in our comparisons. Simulated data often do not reflect error profiles of Illumina reads. All data sets used in this paper have been obtained from the NCBI SRA and have been use previously in [4, 5]. These data sets represent typical data sets that one may use for *de novo* genome assembly.

We evaluate the error-correction software by error correcting the reads of three organisms, two bacterial genomes and a mammalian genome, for which a high quality reference assembly is available: *Rhodobacter sphareoides* (rhodobacter) [21], SRA accession SRR081522 randomly sampled to 45 × genome coverage; *Staphylococcus aureus* (staphylococcus) SRA accession SRR022868, randomly sampled to 45 × genome coverage; and *Mus musculus* (mouse) [22] chromosome 16. The rhodobacter and staphylococcus data sets have previously been used in the GAGE project [5]. At the request of a referee, we added in supplementary material a more recent (2012) data set for *R. sphareoides*, SRX264781. The results are significantly better for this new data set but the relative performance of the correctors remains unchanged. See Supplementary Material S2 Table. For the mouse chromosome 16 data set we downloaded the paired-end reads from NCBI SRA study Mouse_B6_Genome_on_Illumina. These sequences were generated from mouse strain C57BL/6J, the same strain used for the finished mouse sequence [22]. We then mapped the reads to the finished sequence for the entire mouse genome using Bowtie2 [23], and then extracted the reads whose best hit either for the read or for its mate was in chromosome 16. These genomes present different type of challenges for error reduction. The rhodobacter genome (4.6Mb long) has a high GC content and is consequently difficult to sequence using Illumina technology. The staphylococcus genome is 2.9Mb long. The mouse chromosome is larger and has a more complex repeat structure.

We were able to successfully run all error correctors on the bacterial data sets. No results are reported for Echo and HiTec on the mouse due to excessive runtime on larger data sets (see Table 5). In addition, we implemented two simple programs that only trim the input reads. ***trim20B*** trims 20 bases from the 3’ end of the reads, while ***trimQual5*** trims the 3’ end of a read at a base where the quality goes below or equal to 5 and subsequently never goes above 5. When applicable, we also compare the results with making no changes and/or no trimming at all, mentioned in the result tables as the ***none*** corrector. The commands used to run the error correctors are available in Supplementary Material S1 Text.

### False and missing *k*-mers

A *k*-mer is considered ***false*** if it is present in the corrected reads and not in the reference. Note that a false *k*-mer present in multiple reads is counted as 1 false *k*-mer. Conversely, a *k*-mer is ***missing*** if it is present in the reference and not in the corrected reads.

In Table 1 the ***False remain*** is the number of false 31-mers left in the corrected reads as a percentage of the false 31-mers in the original reads. We examined other *k*-mer sizes (*k* = 21,51) and found that the results are similar. See Supplementary Material S3 Table. By definition, the “none error” corrector has 100% of its false 31-mers remaining. The ***True missing*** is the number of missing 31-mers as a percentage of the number of 31-mers in the reference. The numbers of true 31-mers missing from the original reads are 16452, 1047, and 59322 respectively for rhodobacter, staphylococcus, and mouse.

**Table 1:** Percent of false 31-mers remaining and true 31-mers missing in error corrected reads. The numbers for “false remain” and “true missing” in the table are percentages. We list the denominators used for the percentages in the headers of each of these columns. For the “false remain”, this denominator is the number of the false 31-mers in the original reads and for the “true missing”, it is the number of 31-mers in the reference. The “score” *π* = the product of the “false remain” and “true missing” columns. QuorUM’s *π* score is the best with a factor of 30, 15, and 3.5 better than the second best for Rhodobacter, Staphylococcus and Mouse C16 data sets respectively.

Corrector	Rhodobacter			Staphylococcus			Mouse C16		
	False remain (55 M)	True missing (4:6 M)	Score π	False remain (33 M)	True missing (2:9 M)	Score π	False remain (410 M)	True missing (87 M)	Score π
none	100	0.36	40	100	0.037	4	100	0.069	7
trim20B	55	0.39	20	64	0.085	5	50	**0.076**	4
trimQual5	9.4	0.71	7	96	0.039	4	34	0.10	3
Coral	69	0.38	30	56	0.13	7	52	0.22	10
Echo	60	**0.36**	20	55	**0.029**	2	-	-	
HiTec	42	1.1	50	33	0.23	8	-	-	
Quake	8.3	0.71	6	3.3	0.24	0.8	4.6	0.16	0.7
SGA	2.3	1.5	3	0.49	0.61	0.3	7.1	0.16	1
Racer	40	0.93	40	35	0.26	9	30	0.27	8
Musket	40	0.52	20	44	0.067	3	29	0.15	4
QuorUM	**0.29**	0.40	**0.1**	**0.22**	0.087	**0.02**	**2.0**	0.11	**0.2**

### Effectiveness of error reduction

We have found that low counts for both false and missing *k*-mers correlates well with better assembly quality. Intuitively, having many false *k*-mers in a set of reads makes the creation of contigs more difficult for an assembler while missing many *k*-mers leads to a fragmented assembly. In our view, a corrector is effective on a genome if both metrics are small. In either case, QuorUM does better than the other programs on all 3 species. QuorUM consistently has low counts for both the false and missing *k*-mer values while other correctors have less balanced results: either the false or missing *k*-mer value is high. Surprisingly, Echo’s staphylococcus corrected reads have even fewer missing *k*-mers than the original reads (0.029% vs. 0.037%), i.e. it recovers some true *k*-mers which are not present in the original reads. This is possible because Echo does a multiple alignment. But it is not aggressive in its correction and leaves many false *k*-mers. On the other hand, Quake usually leaves few false *k*-mers but is so rigorous in its trimming, that many true *k*-mers are missing from its corrected reads. We introduced a score *π*, which is equal to the product of the “false remain” and “true missing” percentages of *k*-mers listed in Table 1.

This statistic *π* is introduced to measure only how successful an error corrector is at achieving our design goal of minimizing both the “false remain” and “true missing”. The *π* score is introduced for comparing how different error correctors perform on our design idea. For example, if for a given pair of error correctors “false remain” percentages differ by a factor of 2, but “true missing” percentages are the same, then the *π* scores would differ by a factor of two.

Table 2 gives an evaluation of how good the corrected reads might be for creating an assembly. Important metrics of the quality of an assembly include the statistics on the length of the contigs generated, such as the ***N50*** size and the ***E-size*** [5]. In general, the N*x* size is defined as the contig size such that *x*% of the genome is contained in contigs of size N*x* or larger. The expected size, or E-size, of the contigs is computed as follows. For each base (i.e. location) in the reference, compute the size of the contig it lies in. The E-size is the average of these sizes, averaged over all the bases in the finished sequence. In other words, the E-size is the expected contig size for a randomly chosen base in the genome. It is computed as the sum of the squares of the contig lengths divided by the length of the reference assembly.

**Table 2:** Idealized contig size statistics (in kb).

Corrector	Rhodobacter		Staphylococcus		Mouse C16	
	N50	E-size	N50	E-size	N50	E-size
none	2.7	4.1	43	42.4	32	40.4
trim20B	3.9	5.8	17	20.4	32	40.1
trimQual5	3.2	4.3	35	42.3	38	47.8
Coral	4.7	6.9	65	87.7	17	22.6
Echo	5.6	8.0	**100**	110	-	-
HiTec	5.7	8.1	55	56.3	-	-
Quake	3.2	4.3	21	23.1	36	45.1
SGA	4.7	6.8	15	16.2	38	48.1
Racer	5.7	9.3	39	44.3	24	30.2
Musket	5.1	7.8	61	78.1	31	38.6
QuorUM	**12**	**18**	86	**112**	**40**	**49.0**

### Idealized contigs

To avoid any bias in our results related to choosing a particular assembler, we chose to simply map the corrected reads to the reference genome thus creating idealized contigs.

An ***idealized contig*** consists of a segment of the genome that is covered by overlapping corrected reads, overlapping by at least *O* bases (we chose *O* = 25 for reporting the results shown in Table 2 because it corresponds to building an overlap-based assembly with a minimum overlap of 25 bases or de Bruijn graph assembly with 25-mers; one rarely would choose a minimum overlap or smaller *k*-mer size for an assembly). These reads must match the reference along their entire length with at least 98% identity. Given that the idealized contigs are created by alignment to the reference, the N50 and E-size of idealized contigs represent an estimate of the upper-bound of the best N50 and E-size that can be obtained when assembling this set of reads. We note that reads with less than 98% identity will likely be assembled in different contigs by the assembly program. The N50 and E-size of idealized contigs are reported in Table 2.

The correctors that leave too many false *k*-mers in the reads will have fewer reads that align to the reference at 98% identity, resulting in gaps between the idealized contigs. Conversely, too many true *k*-mers missing result in gaps between the idealized contigs. Table 2 suggests that QuorUM generally generates a set of reads that will lead to an assembly with longer contigs.

### Perfect reads

Table 3 reports the percentage of the original reads that are perfect after error reduction, and it also reports the percentage of bases contained in perfect reads compared with bases in original reads. A ***perfect read*** is defined as read having a full length error-free alignment with the reference.

**Table 3:** Percentage of the original reads that are perfect after error reduction, and percentage of bases contained in perfect reads compared with bases in original reads. The number in parenthesis is the denominator used to compute the percentage, the number of original reads and the amount of sequence in the original reads respectively.

Corrector	Rhodobacter		Staphylococcus		Mouse C16	
	Reads (2 M)	Sequence (202 M)	Reads (1:2 M)	Sequence (120 M)	Reads (41 M)	Sequence (4:2 G)
none	21	21	33	33	48	48
trim20B	44	36	46	37	79	64
trimQual5	76	51	35	35	78	72
Coral	58	58	74	74	81	81
Echo	56	56	65	65	-	-
HiTec	61	61	78	78	-	-
Quake	81	59	69	60	89	81
SGA	62	62	75	75	85	85
Racer	63	63	78	78	84	84
Musket	76	70	80	78	88	86
QuorUM	**90**	**77**	**84**	**81**	**92**	**88**

We can use these numbers to determine the average length of the corrected reads as a fraction of their original length. For example, consider rhodobacter. Of the QuorUM corrected reads, 90% are perfect and they contain 77% of the bases in all of the original reads. It follows that the perfect reads have average length 77/90 = 0.86 times the original read length. Hence, 14% of the bases those reads were trimmed away. The non-trimming correctors, by definition, have the same value for both columns. By comparison, Quake trimmed away 28% and Musket trimmed away 8%.

QuorUM consistently produces the most sequence in perfect reads. At the request of a referee, we have included as supplementary material S1 Fig. three graphs, one for each of the three species we study. We plot the percentage (that is the percentage of the uncorrected reads) of reads that are perfect (full read length no error match) versus a minimum read length. For lengths close to 100% of the original read length the non-trimming error correctors perform better in these plots.

### Chimeric reads

We call a read ***chimeric*** when it merges together sequences from two or more distant regions of the genome. We declare a read to be chimeric when it matches in two disjoint pieces in the genome better than any single match of the whole read. Specifically, using Bowtie [23], a read is chimeric if satisfies two conditions. First, there is no match in one piece at 98% identity. Second it can be divided into two non-overlapping pieces (each at least 20 bases long) that match in two disjoint places at 98% identity.

Table 4 reports the effect of error reduction on chimeric reads. Such reads typically cannot be corrected and have to be trimmed or discarded.

**Table 4:** Number of chimeric reads per 10000 after correction.

Corrector	Rhodobacter	Staphylococcus	Mouse C16
none	11	7.3	59
trim20B	7.9	4.9	46
trimQual5	2.9	7.1	26
Coral	11	9.3	52
Echo	9.6	7.6	-
HiTec	35	12	-
Quake	**0.100**	**5.2**	**11**
SGA	3.8	5.7	14
Racer	18	8.8	65
Musket	15	8.6	40
QuorUM	0.17	7.2	13

The Illumina technology generates few chimeric reads (usually much less than 1% of all the reads). When aggressively changing bases, one runs the risk of creating new chimeric reads; i.e. sequence from a distant, possibly repeated, region of the genome may be used to rewrite significant portion of a read.

Quake performs best at reducing the number of chimeric reads, although, this often comes at the cost of very aggressive trimming. Musket and HiTec increase the number of chimeric reads.

### Computation time of the error corrector programs

**Fig 1:**
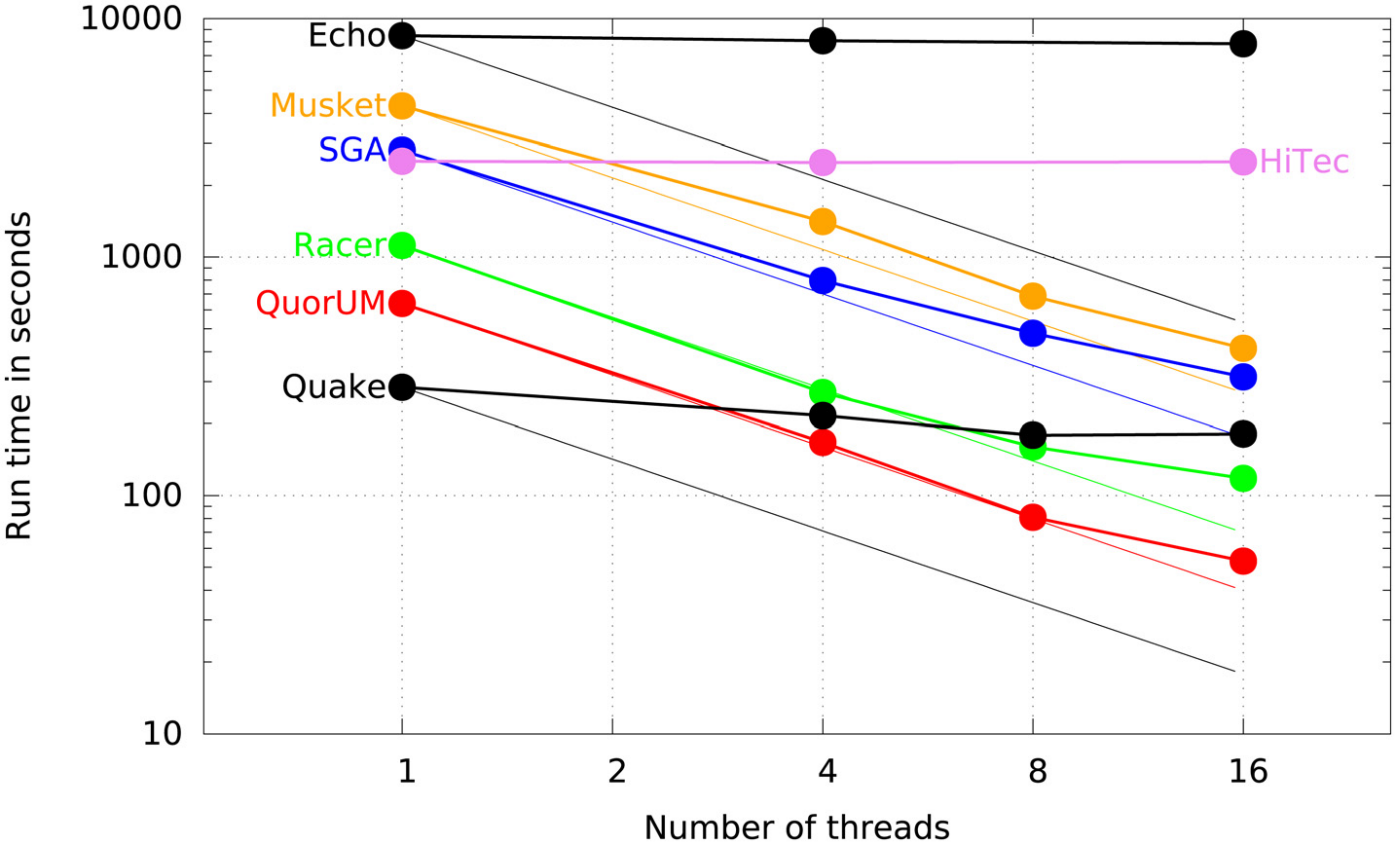
Runtime of the error corrector programs on Rhodobacter vs. the number of threads. The solid lines represent the actual runtime while the dashed lines represent the perfect linear speed-up (except for HiTec which is not multi-threaded). The plot uses a log-log scale.

Table 5 reports the runtime to error correct reads for each program, on all three data sets using 16 AMD 8389 cores. QuorUM is significantly faster than all the other programs. Fig 1 shows the runtime on Rhodobacter while varying the number of threads used. To measure the runtime with different number of threads, we use the ‘numactl’ command of Linux to restrict at the system level the number of execution cores available to the programs. HiTec is not multi-threaded and it does not benefit from the availability of multiple cores. Echo is multi-threaded, but it is written in Python, and the Python interpreter does not scale over multiple cores, thus the speed-up is limited. The other programs benefit from multiple cores, with Racer and QuorUM having a speed-up closest to the theoretical linear limit. Using 16 threads QuorUM is significantly faster than the other error correctors on all data sets.

**Table 5:** Runtime of each program in hours:minutes:seconds, using 16 threads, and memory usage in giga-bytes. The number of bases in each genome is reported in each column.

Corrector	Rhodobacter 4:6Mb		Staphylococcus 2:9Mb		Mouse C16 98:2Mb	
	Time	Mem	Time	Mem	Time	Mem
Coral	0:09:46	35	0:06:18	33	-	-
Echo	2:10:46	58	1:06:11	39	-	-
HiTec	0:41:51	4.0	0:22:09	2.3	-	-
Quake	0:03:01	0.37	0:04:18	1.3	1:13:30	5.7
SGA	0:05:14	0.34	0:03:23	0.28	0:32:33	2.1
Racer	0:01:58	2.0	0:01:01	1.4	0:34:35	11
Musket	0:06:54	**0.23**	0:01:49	**0.22**	0:58:11	**0.38**
QuorUM	**0:00:53**	0.44	**0:00:17**	0.74	**0:23:12**	8.8

QuorUM was used in error correcting the 15 billion read data set used for assembling the 2Gb Loblolly Pine genome [24]. The peak memory usage was 480Gb.

### Impact of error-correction on assembly

To illustrate the utility of using QuorUM to pre-process Illumina data before running a *de novo* assembly, we assembled the three data sets used in this paper. We used two assemblers: SOAPdenovo2 [25] version 240, which is a popular assembler for Illumina data, and MaSuRCA [7], an assembler designed to use QuorUM corrected reads. The results of the SOAPdenovo assemblies provide an unbiased comparison between the correctors as SOAPdenovo is developed independently from any of the error correctors. On the other hand, the MaSuRCA assemblies are of better quality and using MaSuRCA is the recommended way to use QuorUM corrected reads. For SOAPdenovo, we used the recommended assembler parameters from http://soap.genomics.org.cn/soapdenovo.html with *k* = 31. SOAPdenovo was run twice with the settings “-d0” and “-d1”. The former instruct SOAPdenovo to use all *k*-mers, while the latter instruct to ignore *k*-mers with count 1. In itself, the setting “-d1” can be viewed as a crude error reduction technique: most count 1 *k*-mers are indeed errors as we discussed above.

In Table 6 we show the contigs’ ***NGA50*** for the various error-correctors used with SOAPdenovo. For QuorUM we list the MaSuRCA assembly NGA50 in parenthesis. The complete results for all error-correctors with both assemblers are provided in Supplementary Material S1 Table. The NGA50, computed using Quast [26] version 2.3, is the N50 with respect to the finished genome size of the contig sizes after being split at misassemblies. Quast only uses contigs of at least 500 bases for computing NGA50. A reported value of 0. means that the amount of sequence in the split contigs of size greater than 500 bases is less than half of the reference sequence.

**Table 6:** The assembled NGA50 contig size in kilo-bases for SOAPdenovo. The “-d0” and “-d1” are parameters to SOAPdenovo instructing the assemblers to use all 31-mers or to ignore the 31-mers occurring only once. For MaSuRCA, which incorporates QuorUM, the result is in parentheses.

Corrector	Rhodobacter		Staphylococcus		Mouse C16	
	-d0	-d1	-d0	-d1	-d0	-d1
none	0.	2.7	0.	4.8	0.64	1.5
Coral	0.	3.4	0.67	16	-	-
Echo	0.	3.1	0.92	9.1	-	-
HiTec	0.96	2.3	3.0	8.5	-	-
Quake	2.9	1.6	10	5.7	**1.7**	**1.7**
SGA	3.4	2.3	8.4	5.9	1.4	**1.7**
Racer	1.2	2.5	5.2	7.8	1.2	1.4
Musket	0.53	2.9	2.1	9.5	1.2	1.4
QuorUM	**6.6**	5.9	**19**	16.206	**1.7**	**1.7**
MaSuRCA	(19)		(33)		(5.7)	

QuorUM improves SOAPdenovo assemblies more than the other error-correctors. Using QuorUM with MaSuRCA yields even better results.

## Conclusion

Our algorithm for correcting reads focuses on achieving our design idea of minimizing the number of distinct erroneous *k*-mers in the output reads, while preserving the most true *k*-mers. The algorithm is admittedly complex due to the complex nature of Illumina read data. It has been and remains our view that if we achieve this design idea, the *π* score will be low, and the QuorUM corrected reads will produce better assemblies. To assist the reader in evaluating how well we achieve this design idea we have created the statistic *π*, and we show the values of *π* in Table 1. The values of *π* are lowest for QuorUM by factors of 30, 15, and 3.5. Of course this reflects that the design ideas of the other correctors are different, not aimed at minimizing *π*. But, as we argue below, our reads are better for assemblies, and we leave it to the reader to determine if the low scores of *π* are the reason why our assemblies are good.

From the point of view of using error correctors as aids in the assembly of whole genome shotgun reads, the most revealing criterion is the size of the idealized contigs (Table 2). We also illustrate that error correction with QuorUM can benefit in assembly using an independent third-party assembly tool that has not been optimized for use with QuorUM (Table 6). QuorUM produces a larger proportion of error-free reads (Table 3) and its reads yield the largest idealized contig size (Table 2). Chimeric reads can result in misassemblies of contigs and Quake is best at eliminating chimeric reads. Also, QuorUM is the fastest error corrector according to our timing statistics listed in Table 5. Overall, by most criteria in this paper, QuorUM is the best error corrector for the purpose of genome assembly.

## Supporting Information

S1 Text Corrector commands. List of all commands and parameters used to run the error correctors and assemblers.
(PDF)

S1 Table NGA50 and NG50 for SOAPdenovo and MaSuRCA. Additional quality statistics the assemblies generated.
(PDF)

S1 Fig Perfect reads. Perfect reads versus minimum read length.
(PDF)

S2 Table Rhodobacter SRX264781. Extra dataset.
(PDF)

S3 Table Robustness to choice of *k*. False remain and true missing table for different *k*-mer lengths.
(PDF)
