# Supplementary material for: QuorUM: An Error Corrector for Illumina Reads
Source: PLoS One. 2015 Jun 17;10(6):e0130821. doi: 10.1371/journal.pone.0130821 (PMC4471408; doi:10.1371/journal.pone.0130821)
Supplement: S1 Text — List of all commands and parameters used to run the error correctors and assemblers. (PDF) [file pone.0130821.s001.pdf]

Corrector commands

The various error correctors were run using a Makefile. In the following code listing, the variable '$<' represents the input file, '$@' represents the output file, '$(NTH)' is the number of CPU cores to use, '$(JF_SIZE)' is an estimate of the number of k-mers in the reads, and '$(GENOME_SIZE)' is the size of the genome. Some of the software below use multiple commands (e.g. Quake and QuorUM). Both of them have easier to use wrapper scripts.

Echo

```
python ErrorCorrection.py --ncpu $(NCPU) -o $@ $<
```

HiTec

```
hitec $< $@ $(GENOME_SIZE) 1
```

QuorUM

```
quorum_create_database -t $(NTH) -s $(JF_SIZE) -b 8 -m 24 -q 37 -o mer_db.jf $<
quorum_error_correct_reads --contaminant share/adapter.jf -m 1 -s 1 -g 1 -a 3 \
  -t $(NTH) -w 10 -e 3 -o quorum/corrected_tmp mer_db.jf $<
```

Musket

```
musket -k 28 $(JF_SIZE) -o $@.tmp -p $(NTH) -maxtrim 50 $<
```

SGA

```
sga index -a ropebwt -t $(NTH) -p prefix $<
sga correct -p prefix -t $(NTH) --discard --metrics metrics -o $@ $<
```

Racer

```
RACER $< $@ $(GENOME_SIZE)
```

Quake For Quake, the file ' fastq_list ' contains the names of the fastq files with paired reads.

```
count-qmers -k 15 -f $< > qmer_counts
cov_model.py qmer_counts > cutoff.log
correct -f fastq_list -k 15 -c `cat cutoff` -m qmer_counts -p $(NTH)
```

Assembler commands

The SOAPdenovo assembler was run as follows:

```
SOAPdenovo-63mer all -s soap.conf -d 0 -K 31 -R -o output -N $(GENOME_SIZE) -p $(NTH)
```

with the following configuration file "soap.conf":

```
#maximal read length
max_rd_len=101
[LIB]
#average insert size
avg_ins=180
#if sequence needs to be reversed
reverse_seq=0
#in which part(s) the reads are used
asm_flags=3
```

```
#use only first 100 bps of each read
rd_len_cutoff=101
#in which order the reads are used while scaffolding
rank=1
# cutoff of pair number for a reliable connection (at least 3 for short insert size)
pair_num_cutoff=3
#minimum aligned length to contigs for a reliable read location (at least 32 for short i
map_len=32
#a pair of fastq file, read 1 file should always be followed by read 2 file
f1=frag_cor_1.fasta
f2=frag_cor_2.fasta
```

To run MaSuRCA, the following configuration file was used:

```
PARAMETERS
GRAPH_KMER_SIZE = auto
CA_PARAMETERS= ovlMerSize=30 cgwErrorRate=0.25 merylMemory=8192 ovlMemory=4GB
LIMIT_JUMP_COVERAGE = 60
KMER_COUNT_THRESHOLD = 1
EXTEND_JUMP_READS=0
NUM_THREADS= 16
JF_SIZE=200000000
DO_HOMOPOLYMER_TRIM=1
USE_LINKING_MATES=1
END

DATA
PE= pe 180 20 frag_1.fastq frag_2.fastq
END
```

Then the resulting “assemble.sh” script was modified to not run QuorUM and instead take already error-corrected reads as input.
