# Supplementary material for: QuorUM: An Error Corrector for Illumina Reads
Source: PLoS One. 2015 Jun 17;10(6):e0130821. doi: 10.1371/journal.pone.0130821 (PMC4471408; doi:10.1371/journal.pone.0130821)
Supplement: S1 Table — Additional quality statistics the assemblies generated. (PDF) [file pone.0130821.s002.pdf]

NGA50 and NG50 for SOAPdenovo and MaSuRCA

In Table 1 and Table 2 are the NGA50 and NG50 for both SOAPdenovo and MaSuRCA assemblies.

Table 1. The assembled NGA50 contig size (in kilo-bases) for SOAPdenovo and MaSuRCA assemblies. The “-d0” and “-d1” are parameters to SOAPdenovo instructing the assemblers to use all 31-mers or to ignore the 31-mers occurring only once.

Corrector	Rhodobacter			Staphylococcus			Mouse C16			R. SRX264781		
	SOAPdenovo		MaSuRCA	SOAPdenovo		MaSuRCA	SOAPdenovo		MaSuRCA	SOAPdenovo		MaSuRCA
	-d0	-d1		-d0	-d1		-d0	-d1		-d0	-d1	
none	0.	2.7	0.	0.	4.8	0.	0.64	1.5	2.7	0.	15	0.
Coral	0.	3.4	0.	0.67	**16**	2.1	-	-	-	0.	**29**	0.
Echo	0.	3.1	0.	0.92	9.1	1.2	-	-	-	1.1	22	0.
HiTec	0.96	2.3	0.	3.0	8.5	6.5	-	-	-	2.4	13	60
Quake	2.9	1.6	5.0	10	5.7	16	**1.7**	**1.7**	**6.2**	8.8	6.9	53
SGA	3.4	2.3	3.4	8.4	5.9	10	1.4	1.4	5.2	20	15	61
Racer	1.2	2.5	0.	5.2	7.8	4.2	1.2	1.2	6.0	4.5	15	52
Musket	0.53	2.9	0.	2.1	9.5	0.	1.2	1.4	5.7	1.4	18	0.
QuorUM	**6.6**	**5.9**	**19**	**19**	16	**33**	**1.7**	**1.7**	5.7	**26**	25	**130**

Table 2. The assembled NG50 contig size (in kilo-bases) for SOAPdenovo and MaSuRCA assemblies. The “-d0” and “-d1” are parameters to SOAPdenovo instructing the assemblers to use all 31-mers or to ignore the 31-mers occurring only once.

Corrector	Rhodobacter			Staphylococcus			Mouse C16			R. SRX264781		
	SOAPdenovo		MaSuRCA	SOAPdenovo		MaSuRCA	SOAPdenovo		MaSuRCA	SOAPdenovo		MaSuRCA
	-d0	-d1		-d0	-d1		-d0	-d1		-d0	-d1	
none	0.	2.7	0.	0.	4.8	0.	0.64	1.5	2.7	0.	16	0.
Coral	0.	3.4	0.	0.67	**17**	2.1	-	-	-	0.	**30**	0.
Echo	0.	3.1	0.	0.92	9.2	1.2	-	-	-	1.1	22	0.
HiTec	0.96	2.3	0.	3.0	8.5	6.5	-	-	-	2.4	13	60
Quake	2.9	1.6	5.0	10	5.7	17	**1.7**	**1.7**	**6.2**	8.8	6.9	54
SGA	3.4	2.3	3.4	8.4	5.9	10	1.4	1.4	5.3	20	15	61
Racer	1.2	2.5	0.	5.2	7.9	4.2	1.2	1.2	6.0	4.5	15	52
Musket	0.53	2.9	0.	2.1	9.5	0.	1.2	1.4	5.7	1.4	18	0.
QuorUM	**6.6**	**5.9**	**19**	**19**	16	**33**	**1.7**	**1.7**	5.8	**26**	25	**130**
