# Supplementary material for: QuorUM: An Error Corrector for Illumina Reads
Source: PLoS One. 2015 Jun 17;10(6):e0130821. doi: 10.1371/journal.pone.0130821 (PMC4471408; doi:10.1371/journal.pone.0130821)

Perfect reads

Percentage (that is the percentage of the uncorrected reads) of reads that are perfect (full read length no error match) versus a minimum read length, for Rhodobacter (Figure 1), for Staphylococcus (Figure 2) and for Mouse C16 (Figure 3).

Figure 1. Percentage of perfect reads vs. minimum length for Rhodobacter.

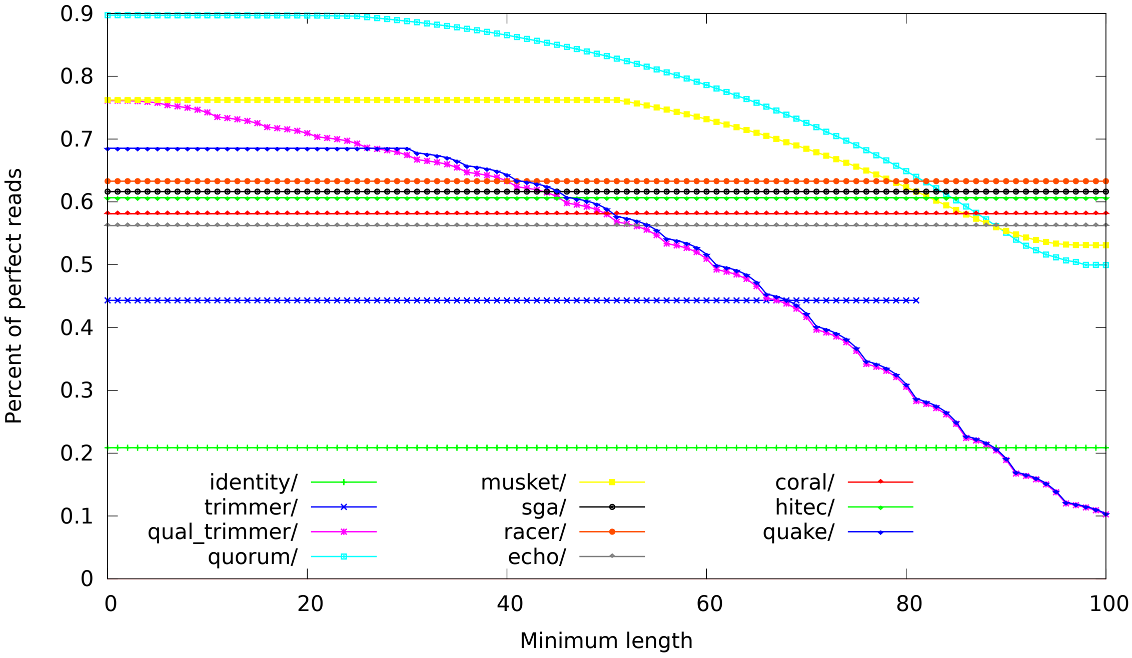

Figure 2. Percentage of perfect reads vs. minimum length for Staphylococcus.

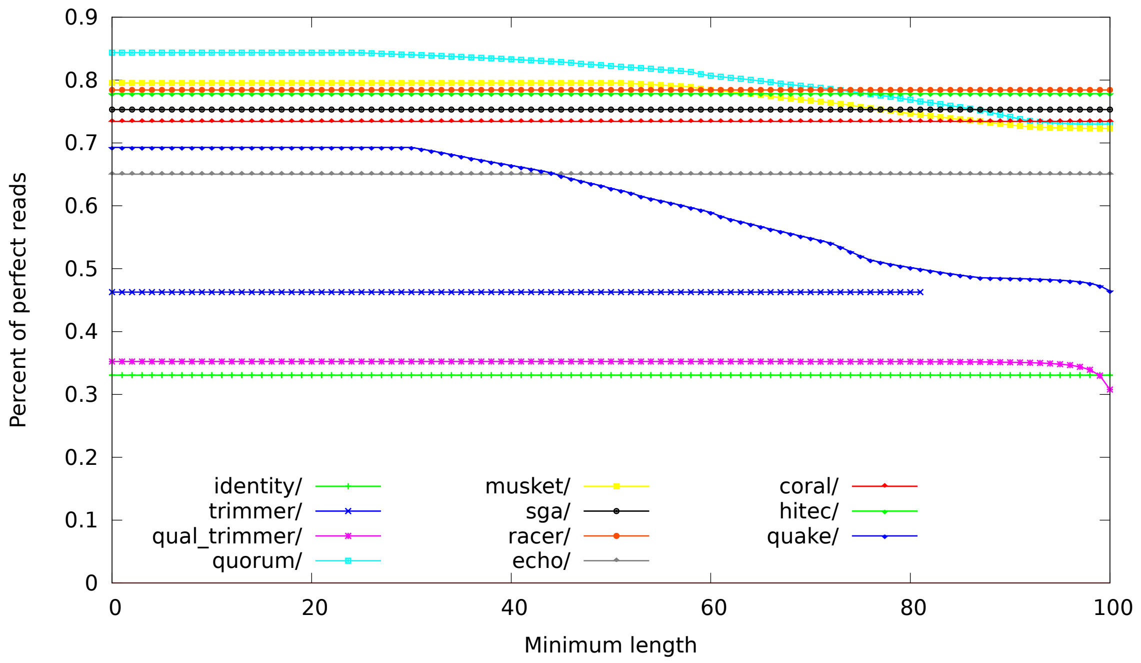

Figure 3. Percentage of perfect reads vs. minimum length for Mouse C16.

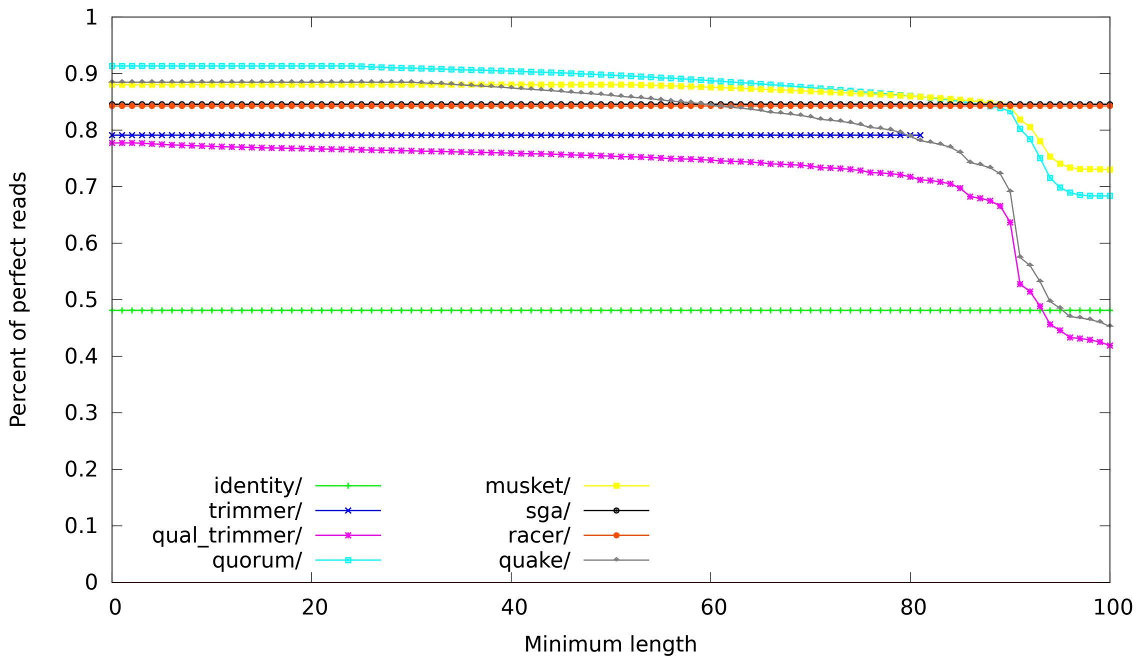

Supplement: S1 Fig — Perfect reads versus minimum read length. (PDF) [file pone.0130821.s003.pdf]
