# Supplementary material for: QuorUM: An Error Corrector for Illumina Reads
Source: PLoS One. 2015 Jun 17;10(6):e0130821. doi: 10.1371/journal.pone.0130821 (PMC4471408; doi:10.1371/journal.pone.0130821)
Supplement: S2 Table — Extra dataset. (PDF) [file pone.0130821.s004.pdf]

# Rhodobacter SRX264781

The tables 1, 2, 3, 4, and 5 are similar to the tables in the main text, with the addition of *R. sphareoides* accession SRX264781 data set. This extra data set is the same strand as the other *R. sphareoides* data set but is a PCR free library and was sequenced more recently (2012 vs. 2010). The relative performance of QuorUM versus the other error corrector is identical on this more recent data set.

**Table 1. Percent of false 31-mers remaining and true 31-mers missing in error corrected reads.**

| Corrector | Rhodobacter | | | Staphylococus | | | Mouse C16 | | | R. SRX264781 | | |
|---|---|---|---|---|---|---|---|---|---|---|---|---|
| | False remain (55 M) | True missing (4.6 M) | Score $\pi$ | False remain (33 M) | True missing (2.9 M) | Score $\pi$ | False remain (410 M) | True missing (87 M) | Score $\pi$ | False remain (48 M) | True missing (4.6 M) | Score $\pi$ |
| none | 100 | 0.36 | 40 | 100 | 0.037 | 4 | 100 | 0.069 | 7 | 100 | 0.15 | 20 |
| trim20B | 55 | 0.39 | 20 | 64 | 0.085 | 5 | 50 | **0.076** | 4 | 64 | 0.16 | 10 |
| trimQual5 | 9.4 | 0.71 | 7 | 96 | 0.039 | 4 | 34 | 0.10 | 3 | 19 | 0.19 | 4 |
| Coral | 69 | 0.38 | 30 | 56 | 0.13 | 7 | 52 | 0.22 | 10 | 49 | 0.33 | 20 |
| Echo | 60 | **0.36** | 20 | 55 | **0.029** | 2 | - | - | - | 38 | **0.15** | 6 |
| HiTec | 42 | 1.1 | 50 | 33 | 0.23 | 8 | - | - | - | 26 | 0.22 | 6 |
| Quake | 8.3 | 0.71 | 6 | 3.3 | 0.24 | 0.8 | 4.6 | 0.16 | 0.7 | 9.0 | 0.18 | 2 |
| SGA | 2.3 | 1.5 | 3 | 0.49 | 0.61 | 0.3 | 7.1 | 0.16 | 1 | 0.53 | 0.22 | 0.1 |
| Racer | 40 | 0.93 | 40 | 35 | 0.26 | 9 | 30 | 0.27 | 8 | 25 | 0.24 | 6 |
| Musket | 40 | 0.52 | 20 | 44 | 0.067 | 3 | 29 | 0.15 | 4 | 36 | 0.18 | 6 |
| QuorUM | **0.29** | 0.40 | **0.1** | **0.22** | 0.087 | **0.02** | **2.0** | 0.11 | **0.2** | **0.24** | 0.16 | **0.04** |

**Table 2. Idealized contig size statistics (in kb).**

| Corrector | Rhodobacter | | Staphylococcus | | Mouse C16 | | R. SRX264781 | |
|---|---|---|---|---|---|---|---|---|
| | N50 | E-size | N50 | E-size | N50 | E-size | N50 | E-size |
| none | 2.7 | 4.1 | 43 | 42.4 | 32 | 40.4 | 67 | 74.8 |
| trim20B | 3.9 | 5.8 | 17 | 20.4 | 32 | 40.1 | 58 | 77.6 |
| trimQual5 | 3.2 | 4.3 | 35 | 42.3 | 38 | 47.8 | 36 | 51.8 |
| Coral | 4.7 | 6.9 | 65 | 87.7 | 17 | 22.6 | 100 | 142 |
| Echo | 5.6 | 8.0 | **100** | 110 | - | - | 120 | **171** |
| HiTec | 5.7 | 8.1 | 55 | 56.3 | - | - | 110 | 132 |
| Quake | 3.2 | 4.3 | 21 | 23.1 | 36 | 45.1 | 36 | 57.2 |
| SGA | 4.7 | 6.8 | 15 | 16.2 | 38 | 48.1 | 68 | 94.4 |
| Racer | 5.7 | 9.3 | 39 | 44.3 | 24 | 30.2 | 99 | 128 |
| Musket | 5.1 | 7.8 | 61 | 78.1 | 31 | 38.6 | 90 | 120 |
| QuorUM | **12** | **18** | 86 | **112** | **40** | **49.0** | **140** | 167 |

**Table 3. Percentage of the original reads that are perfect after error reduction, and percentage of bases contained in perfect reads compared with bases in original reads.**

| Corrector | Rhodobacter | | Staphylococcus | | Mouse C16 | | R. SRX264781 | |
|---|---|---|---|---|---|---|---|---|
| | Reads (2 M) | Sequence (200 M) | Reads (1.2 M) | Sequence (120 M) | Reads (41 M) | Sequence (4.2 G) | Reads (2.0 M) | Sequence (200 M) |
| none | 21 | 21 | 33 | 33 | 48 | 48 | 39 | 39 |
| trim20B | 44 | 36 | 46 | 37 | 79 | 64 | 52 | 42 |
| trimQual5 | 76 | 51 | 35 | 35 | 78 | 72 | 68 | 52 |
| Coral | 58 | 58 | 74 | 74 | 81 | 81 | 80 | 80 |
| Echo | 56 | 56 | 65 | 65 | - | - | 79 | 79 |
| HiTec | 61 | 61 | 78 | 78 | - | - | 84 | 84 |
| Quake | 81 | 59 | 69 | 60 | 89 | 81 | 66 | 55 |
| SGA | 62 | 62 | 75 | 75 | 85 | 85 | 81 | 81 |
| Racer | 63 | 63 | 78 | 78 | 84 | 84 | 86 | 86 |
| Musket | 76 | 70 | 80 | 78 | 88 | 86 | 86 | 84 |
| QuorUM | **90** | **77** | **84** | **81** | **92** | **88** | **90** | **86** |

**Table 4. Number of chimeric reads per 10 000 after correction.**

| Corrector | Rhodobacter | Staphylococcus | Mouse C16 | R. SRX264781 |
|---|---|---|---|---|
| none | 11 | 7.3 | 59 | 2.0 |
| trim20B | 7.9 | 4.9 | 46 | 1.5 |
| trimQual5 | 2.9 | 7.1 | 26 | 0.81 |
| Coral | 11 | 9.3 | 52 | 1.7 |
| Echo | 9.6 | 7.6 | - | 1.3 |
| HiTec | 35 | 12 | - | 6.8 |
| Quake | **0.100** | **5.2** | **11** | 0.68 |
| SGA | 3.8 | 5.7 | 14 | 0.56 |
| Racer | 18 | 8.8 | 65 | 2.3 |
| Musket | 15 | 8.6 | 40 | 1.7 |
| QuorUM | 0.17 | 7.2 | 13 | **0.24** |

**Table 5. Runtime of each program in hours:minutes:seconds, using 16 threads, and memory usage in giga-bytes.**

| Corrector | Rhodobacter 4.6 Mb | | Staphylococcus 2.9 Mb | | Mouse C16 98.2 Mb | | R. SRX264781 4.6 Mb | |
|---|---|---|---|---|---|---|---|---|
| | Time | Mem | Time | Mem | Time | Mem | Time | Mem |
| Coral | 0:09:46 | 35 | 0:06:18 | 33 | - | - | 0:08:24 | - |
| Echo | 2:10:46 | 58 | 1:06:11 | 39 | - | - | 2:02:37 | - |
| HiTec | 0:41:51 | 4.0 | 0:22:09 | 2.3 | - | - | 0:37:30 | - |
| Quake | 0:03:01 | 0.37 | 0:04:18 | 1.3 | 1:13:30 | 5.7 | 0:03:01 | - |
| SGA | 0:05:14 | 0.34 | 0:03:23 | 0.28 | 0:32:33 | 2.1 | 0:04:07 | - |
| Racer | 0:01:58 | 2.0 | 0:01:01 | 1.4 | 0:34:35 | 11 | 0:01:27 | - |
| Musket | 0:06:54 | **0.23** | 0:01:49 | **0.22** | 0:58:11 | **0.38** | 0:03:09 | - |
| QuorUM | **0:00:53** | 0.44 | **0:00:17** | 0.74 | **0:23:12** | 8.8 | **0:00:30** | - |
