# Supplementary material for: QuorUM: An Error Corrector for Illumina Reads
Source: PLoS One. 2015 Jun 17;10(6):e0130821. doi: 10.1371/journal.pone.0130821 (PMC4471408; doi:10.1371/journal.pone.0130821)
Supplement: S3 Table — False remain and true missing table for different k-mer lengths. (PDF) [file pone.0130821.s005.pdf]

Robustness to choice of k

Tables 1 and 2 are equivalent to Table 1 in the main text, generated for a difference values for the length of the k-mers. The relative performance of QuorUM versus the other error corrector is identical for all these choice of k.

Table 1. Percent of false 21-mers remaining and true 21-mers missing in error corrected reads.

Corrector	Rhodobacter			Staphylococus			Mouse C16			R. SRX264781		
	False remain (51 M)	True missing (4.6 M)	Score π	False remain (32 M)	True missing (2.9 M)	Score π	False remain (360 M)	True missing (87 M)	Score π	False remain (46 M)	True missing (4.6 M)	Score π
none	100	0.17	20	100	0.0097	1.0	100	0.017	2	100	0.11	10
trim20B	57	0.17	10	67	0.017	1	51	**0.020**	1	67	0.11	7
trimQual5	8.3	0.29	2	96	**0.010**	1.0	29	0.034	1	19	0.12	2
Coral	70	0.18	10	62	0.050	3	-	-	-	54	0.18	10
Echo	60	**0.17**	10	57	0.010	0.6	-	-	-	40	**0.11**	4
HiTec	40	0.60	20	37	0.13	5	-	-	-	29	0.15	4
Quake	7.2	0.30	2	2.9	0.15	0.4	2.9	0.090	0.3	8.4	0.12	1
SGA	1.4	1.1	1	0.31	0.48	0.1	3.8	0.087	0.3	0.38	0.16	0.06
Racer	39	0.45	20	40	0.12	5	29	0.093	3	29	0.15	4
Musket	43	0.24	10	49	0.024	1	30	0.046	1	41	0.12	5
QuorUM	**0.30**	0.23	**0.07**	**0.19**	0.038	**0.007**	**1.1**	0.040	**0.04**	**0.21**	0.12	**0.02**

Table 2. Percent of false 51-mers remaining and true 51-mers missing in error corrected reads.

Corrector	Rhodobacter			Staphylococus			Mouse C16			R. SRX264781		
	False remain (50 M)	True missing (4.6 M)	Score π	False remain (28 M)	True missing (2.9 M)	Score π	False remain (410 M)	True missing (87 M)	Score π	False remain (43 M)	True missing (4.6 M)	Score π
none	100	1.7	200	100	0.42	40	100	0.28	30	100	0.32	30
trim20B	48	2.1	100	55	1.4	80	43	0.35	10	55	0.49	30
trimQual5	9.9	3.7	40	96	0.44	40	37	0.38	10	19	0.55	10
Coral	66	1.5	100	50	0.42	20	-	-	-	43	0.68	30
Echo	58	1.4	80	53	**0.20**	10	-	-	-	36	0.26	9
HiTec	44	2.3	100	31	0.48	10	-	-	-	24	0.36	9
Quake	9.1	3.6	30	3.8	0.62	2	6.2	0.42	3	9.5	0.51	5
SGA	3.8	2.5	9	0.92	0.97	0.9	13	0.37	5	0.85	0.34	0.3
Racer	42	2.1	90	32	0.61	20	35	0.57	20	23	0.40	9
Musket	37	1.5	60	38	0.26	10	29	0.42	10	30	0.31	9
QuorUM	**0.33**	**0.94**	**0.3**	**0.32**	0.24	**0.08**	**4.1**	**0.30**	**1**	**0.33**	**0.25**	**0.08**
